# Nanotechnology-Based Dressings for Wound Management

**DOI:** 10.3390/ph15101286

**Published:** 2022-10-19

**Authors:** Janaína A. Ataide, Beatriz Zanchetta, Érica M. Santos, Ana Laura M. Fava, Thais F. R. Alves, Letícia C. Cefali, Marco V. Chaud, Laura Oliveira-Nascimento, Eliana B. Souto, Priscila G. Mazzola

**Affiliations:** 1 School of Medical Sciences, University of Campinas (UNICAMP), Campinas 13083-888, Brazil; 2 Department of Pharmaceutical Technology, Faculty of Pharmacy, University of Coimbra (UC), Coimbra 3000-548, Portugal; 3 Faculty of Pharmaceutical Sciences, University of Campinas (UNICAMP), Campinas 13083-871, Brazil; 4 Laboratory of Biomaterials and Nanotechnology, University of Sorocaba (UNISO), Sorocaba 18023-000, Brazil; 5 Institute of Biology, University of Campinas (UNICAMP), Campinas 13083-862, Brazil; 6 Center for Biological and Health Sciences, Mackenzie Presbyterian University, Sao Paulo 01302-907, Brazil; 7 Department of Pharmaceutical Technology, Faculty of Pharmacy, University of Porto, Rua de Jorge Viterbo Ferreira, No. 228, 4050-313 Porto, Portugal; 8 REQUIMTE/UCIBIO, Faculty of Pharmacy, University of Porto, Rua de Jorge Viterbo Ferreira, nº. 228, 4050-313 Porto, Portugal

**Keywords:** wound healing, atopic dermatitis, modern dressings, biomaterials, nanotechnology

## Abstract

Wound healing is known to be a complicated and intricate process and commonly classified as chronic or acute. Patients with chronic wounds are of public health concern, and require more attention onto skin lesions, including atopic dermatitis. Despite being a natural process, healing can be impaired by existing chronic de diseases such as diabetes, for example. Recently, wound dressings based in nanotechnology systems have emerged as a viable option to improve the healing process. Current advances in nanotechnology-based systems to release growth factors and bioactive agents represent a great opportunity to develop new therapies for wound treatments. It is essential that healthcare professionals understand the key processes involved in the healing cascade, to maximize care with these patients and minimize the undesirable outcomes of non-healing wounds. Therefore, this review aims to summarize the healing process phases and provide a general overview of dressings based in nanotechnology using biomaterials for the release of active agents in wound site.

## 1. Introduction

The formation of a wound is the result of a disruption of skin integrity, or mucosal surfaces, or organ tissue [1], that have a common repair mechanism despite varying types of skin injury. Wound healing is a regular biological process in the human body, once human skin can promote self-regeneration after damage [2,3].

Despite being natural process, healing comprises a cascade of physiological events [4], with intricate nature, which makes it remarkable how often it occurs without complications [1].

However, this body capacity is dependent on many known factors, such as patient’s underlying health and nutritional status [5], and can be compromised under specific conditions, such as diabetes, non-healing ulcers, extensive skin loss, and deep burns [3,6]. An inappropriate healing process leads to a chronic wound state, with increased infection risk, affecting patient’s health and quality of life [2]. Chronic wounds are also associated with potential morbidity and mortality as well as poor cosmetic outcome [1,7].

Although it is difficult to quantify the economic effects of chronic wounds, some estimations have been performed. In the USA, it is estimated that wound related problems incur an annual expenditure exceeding one billion dollars [1,8]. A more recent study showed that chronic wounds represent a significant cost to self-funded individuals in Australia, and participants in this study spent on average more than 2000 Australian dollars only on wound dressings [9]. Therefore, to minimize patient morbidity and optimize costs, it is essential that healthcare professionals understand the key physiological processes involved in healing [1].

Thus, wound treatment should enhance natural wound healing process, which might require dressings. The development of new technologies can guarantee an effective and efficient healing, thus reducing healing time and recurrence [10]. Materials used for wound dressing should enable all phases of wound healing process, as well as protecting the wound from infection and excessive moisture loss [11].

Nowadays, contamination is the most challenging subject in wound care. To overcome the problem, researches have been intensified and antimicrobial wound dressings have demonstrated promising results in prevention of contamination [12]. Those dressings were designed in various forms using different biomaterials [13]. Among the available dressings, hydrogels have gained considerable attention owing to their properties, and more importantly, easy wound management. Pinese, et al. [14] classified as “smart dressing” the dressings that combine this physical function, wound healing properties, with other substances, such as anti-inflammatory [15], antimicrobial [14,16], bioactivities [17,18], or growth factor [10,17].

The development of nanotechnology-based systems has aroused great interest, mainly for biomedical and pharmaceutical applications for preventing or treating diseases, including wound healing [19,20,21]. Besides nanoparticles, nanotechnology-based delivery systems also include nanofibers, hydrogels, hydrocolloids, and more recently nanohybrids, which are a combination of different nanotechnological systems [22,23]. When materials are decreased to a nanometric size, their surface area and ratio surface area to volume dramatically increase, leading to advanced physicochemical properties [19]. Therefore, nanomaterials can act in wound healing by carrying and delivering therapeutic agents in the wound bead or due to their inherent properties [20].

Thus, this review aims to summarize relevant and overlapping phases of the healing process and provide a general overview of dressings based in nanotechnology using biomaterials for the release of active agents in the wound site.

## 2. Physiology of Wound Healing

According to the Wound Healing Society, a wound is the disarrangement of natural anatomic structure and function [24] that can be classified as acute or chronic. Acute wounds are typically tissue injuries that heal within the expected period. On the other hand, chronic wounds are tissue lesions that heal slowly due to repeated tissue damage and/or other patient’s pathophysiology that interferes with expected timeline or healing cascade [4,24].

**Figure 1:**
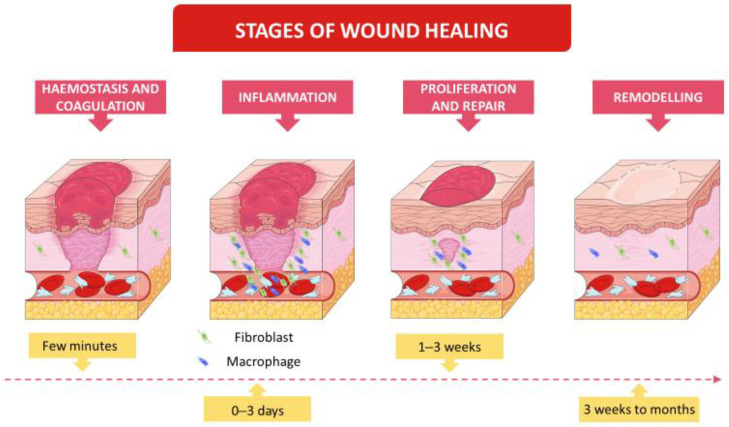
Stages of normal wound healing. The timeline represents body response to injury during a normal healing process, without impairments. Figure modified with text, and cells after adaptation of “Healing” from Servier Medical Art by Servier, licensed under a Creative Commons Attribution 3.0 Unported License.

Healing initiates in response to an injury, with the aim to restore the function and integrity of damaged tissue, and consequently homeostasis [25,26,27,28,29]. The Wound Healing Society defines it as an intricate, dynamic sequence that ends in restoring anatomic continuity as well as function [24]. The normal process (Figure 1) comprises four overlapping phases [4,27,30,31], regulated by cellular, humoral, and molecular mechanisms [29], which will be described hereafter.

Complete wound healing is only possible when all stages occur in the correct sequence, at the specific time, and immunological/biological systems participate in a coordinated way [3,32,33]. In the first three weeks of the healing process, wounds gain only about 20% of skin final strength [4]. Tensile strength could increase from about 20% to a maximum of 70–80% during remodelling phase [34]. Therefore, although the skin appears intact, the tissue underneath is still vulnerable to damage as it passes through the final stages of wound healing [4,29,35].

The strength increase in the final healing stage is due to a slower rate of collagen deposition and, more importantly, to collagen reshaping with the formation of larger bundles of collagen and an increase in intermolecular crosslinks [36]. However, wounds never reach the same breaking strength (the stress at which skin breaks) as uninjured skin. At maximum strength, healed skin would only reach about 80% of original tensile strength [37,38,39].

Not only tensile strength, but certain skin components also never fully recover after wound closure. This is the case of subepidermal appendages as hair follicles and sweat glands, that could not heal or grow back after serious injury. The resulting scar epidermis after healing differs from non-injured skin due to the lack of rete pegs, which are normally anchored in the underlying connective tissue matrix and are responsible for tight connections between the epidermis and dermis [29,40]. Hypertrophic scars and keloids may also occur due to excessive scarring [41].

The wounds generally heal without issues. However, some factors (hypoxia, infection, excessive edema and foreign bodies, for example) interrupt the healing cascade, leading to a chronic wound by establishing a cycle of hypoxia, inflammation, necrosis and infection [42]. Studies focusing on chronic wound treatment aim at strategies to enhance wound healing. Moreover, the development of wound dressing has gained a huge academic and clinical impact [12,17,43].

## 3. Dressing for Wound Treatment

In 1962, Winter concluded that moisturized wounds in piglet skin epithelizes two times faster than air exposed wounds [44]. Since then, much has been learned about wound healing mechanisms and factors that affect them [45,46,47,48], dramatically expanding dressing practices. Over the past years, wound dressings developed from crude applications of natural products, including plant herbs, animal fat, and honey, to tissue engineered scaffolds [49], and more than 3000 products have been developed aiming to treat different wound types and targeting different points of the healing cascade [50].

Nowadays, it is known that this physiological process can be accelerated and enhanced by the use of dressing techniques, products, and actives [11,31,45]. Wound management, then, should be used to avoid complications and improve survival of patients with major chronic wounds and burns by decreasing sepsis events [48,51,52]. In a more specific definition, the main functions of a dressing are to prevent bacterial contamination, absorb exudate, and improve wound healing with more rapid reepithelization [14,53].

### 3.1. Dressing’s Characteristics

In the medical community, there is a consensus that to accelerate wound healing process, an ideal wound dressing should present specific characteristics, such as biocompatibility, adequate water vapor permeability, retain moisture to help wound healing, and provide an antibacterial environment [17,54,55,56]. As a result, dressings should also control tissue dehydration, while removing excess exudate without affecting the healing process.

Some authors pointed out some characteristics of an ideal wound dressing material. Those characteristics include: keep the local environment moisturized; have good gas permeability; remove excess exudates; protect wound from exterior contaminations; stop wound desiccation; reduce the tissue necrosis; stimulate new tissue formation; mechanically protect the wound; be easily and comfortably removable, non-toxic, non-allergic, biocompatible, biodegradable, and elastic; reduce pain around wound; not be costly, i.e., commercially viable; and easily sterilized [7,30,53,57,58].

### 3.2. Advanced Therapeutic Dressings

Dressing products can be classified using different criteria depending on their function, employed material used in the production, dressing physical form, and their contact with wound surface, among others [49]. They can be also classified as traditional and modern wound dressings [49,50,59].

Even though traditional wound dressings are less widely used nowadays, they were commonly used in the past and still provide benefits in certain clinical conditions [49]. Gauze is the most popular of the first generation of conventional wound dressings. However, this type of dressing has many disadvantages in comparison with the newer dressing categories. The main disadvantages that can be highlighted include adhesion on the wound surface, painful upon removal, and extravasation of exudate. Thus, this dressing is inefficient to promote healing and avoid bacterial contamination [30,60,61,62].

On the other hand, modern wound dressings have been developed to facilitate wound and not only to cover it [50], and thus it should retain and create a moist environment in wound site [49]. Modern dressings can be divided into passive, interactive, and biological [50].

Similar to traditional dressings, passive dressings are used to cover the wound, however they are non-occlusive [50]. Most modern products are classified as interactive dressings, and their main characteristic is to interact with the wound environment, providing optimal conditions [59]. This second class of materials are semi-occlusive or occlusive, and they are designed to close and promote the restoration of skin function, also acting as a barrier to microorganism contamination [50].

More recently, attention was directed to biological dressings, especially those containing bioactive agents as growth factors [10,14,17,18,33,61]. Biological dressings are manufactured from biomaterials, which play important roles in the wound healing process. These dressings are known for their biocompatibility, biodegradability, and non-toxic nature [49,50].

### 3.3. Biomaterials Used in the Development of Wound Dressings

Biomaterials have a wide variety of interesting characteristics for development of dressings such as biocompatibility and biodegradability, controlled release, high drug-loading and special mechanical properties [17,63]. The choice of biocompatible materials to produce wound dressings is related to the functions and/or specific properties of these materials [49,50].

Natural or synthetic new biodegradable materials have been used for many applications, such as food and cosmetic/pharmaceutical industries, biosensor design and wound dressings. Chitosan, silk fibroin, starch, phospholipids, cellulose, chitin, alginate, gelatin, collagen, natural rubber, hyaluronic acid, and carrageenan composites or blends are some examples of these materials [50,64,65,66,67,68,69,70,71,72,73,74].

Although natural polymers are the most used ones, synthetic polymers have also gained more attention because they exhibit better mechanical properties and have the advantage of easily control physicochemical properties. Among them, polylactic acid (PLA), poly(ε-caprolactone) (PCL), polyhydroxyalkanoates (PHAs), polyglicolic acid (PGA), as well as their combinations, are the most used synthetic polymers. Moreover, some of these polymers have a great biocompatibility and formulations that include both natural and synthetic polymers present an interesting approach to wound dressing development [17,63,75].

**Figure 2:**
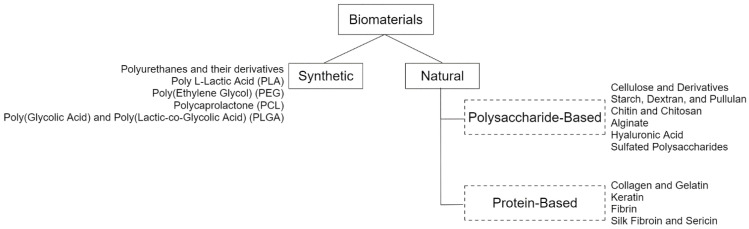
Biomaterials previously used for wound healing classified into synthetic and natural polymers, which have been previously and deepened reviewed elsewhere [79,80,81].

Interactive and biological dressings can be designed in different pharmaceutical forms, such as hydrogels, films, foams, sponges, hydrocolloids, hydrofiber and hydrofilms, which have been previously reviewed [50,59,76,77,78]. Those dressing definitions and main characteristics are summarised in Table 1. In recent literature, biomaterials and their different forms of application have been already reviewed by [79,80,81], as illustrated in Figure 2.

### 3.4. Nanotechnology-Based Delivery Systems for Wound Healing

Nanotechnology is applied in various medical therapies, including the treatment of different types of wounds [19,89]. In recent years, nanotechnology platforms have emerged and nanotechnology-based wound healing therapies are currently under investigation [90]. Nanotechnology platforms, mainly nanoparticles, have been used as novel therapeutic materials to accelerate the wound healing process [91]. Noteworthy, besides the nanoparticles and liposomes, hydrofibers (nanofibers), hydrogel, and hydrocolloids are also classified as nanotechnology-based delivery systems. Currently, hybrid formulations, also called nanohybrid, have shown promising ability to accelerate the wound healing process. Nanohybrid can be defined as a combination of different nanotechnology-based delivery systems, and a classic example is the hydrogel composed of nanoparticles loaded with pharmacological moieties [22,23]. Nanoparticles, polymeric nanofibers, and nanohybrids are discussed in the following sections, and Table 2 summarizes their preparation process.

**Figure 3:**
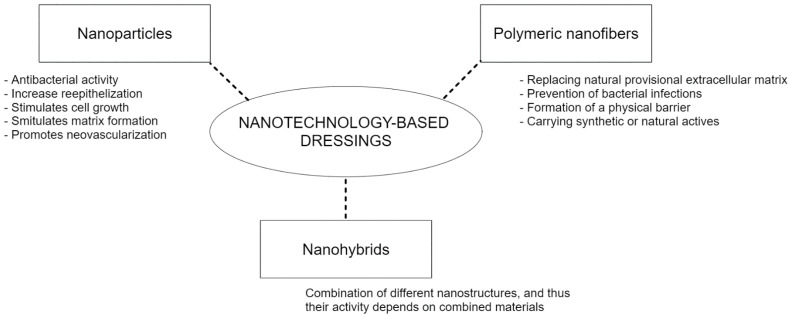
Potential mechanisms of how nanotechnology-based dressings maybe involved in accelerating wound healing, besides of carrying and delivering other actives.

In most cases, nanotechnology-based dressings are used to deliver actives to the wound bead, and then the mechanism by which wound healing is accelerated is dependent on the agent mechanism [19,20,21]. However, reducing the size of materials to the nanoscale leads to changes in their physicochemical properties, which can also influence and accelerate the healing process. Some characteristics that can influence wound healing are biocompatibility, biodegradability, stability, size, as well as surface functionalization and charge [20]. Besides them, other possible mechanisms of how nanotechnology-based dressings can accelerate wound healing [19,104] are shown in Figure 3.

#### 3.4.1. Nanoparticles

Various materials (i.e., polymers, lipids, inorganic materials and their combinations) were used to produce a myriad of nanoparticles with desired physicochemical properties and biological functions [91]. Moreover, nanoparticles have been extensively studied for delivery of a variety range of therapeutic agents, including antibiotics, targeted in treating skin inflammatory diseases [112].

Nanoparticles emerged as a promising strategy to minimize microbial resistance, due their ability to enhance the antimicrobial properties [13], for example it is known that silver nanoparticles demonstrate excellent bacteriostatic and bactericidal activities [19]. Naraginti et al. [92] evaluated the in vivo activity of gold and silver nanoparticles in wound healing. The results show a considerable reduction of healing period, which can be assigned to their antimicrobial and anti-inflammatory properties.

Silver nanoparticles or nanoparticles containing silver have also been studied for wound healing applications [105]. Cotton dress fabrics saturated with silver nanoparticles were compared with fabrics saturated with commercial ointment in the healing of rats’ burn wounds. Nanoparticle fabrics showed a slightly greater healing efficacy, with higher wound contraction area and better fibril alignments in repaired skin [94].

Zinc oxide nanoparticles are known for their antibacterial effect [93,105,113], in vitro adhesion between cells and tissues, and pro-angiogenic properties [95,114,115]. Thus, they have been applied to different materials and formulations, aiming the development of wound healing dressings [106,107,109,111].

Polymeric nanoparticles provide a controlled release of the encapsulated compounds used for wound healing applications [10]. Chitosan nanoparticles were used as drug carriers for silver sulfadiazine, presenting continuous delivery of antibiotic over 24 h, which was higher than the delivery of commercial product (two hours). It also presented proven effectivity for Gram-positive (*Bacillus subtilis* and *Staphylococcus aureus*) and Gram-negative (*Escherichia coli* and *Pseudomonas aeruginosa*) bacteria and *Candida albicans* on an infected wound [96]. Chitosan has also been used to encapsulate bromelain, a proteolytic enzyme that can be used in wound debridement, aiming to enhance its stability [97,98,99].

Among lipid nanoparticles, liposomes present the ability to increase drug accumulation in the skin, which contributes for wound healing and atopic dermatitis [10,116,117]. Phospholipids are commonly used to develop liposomes and lipid nanoparticles. Lipids exhibit biocompatibility and biodegradability, controlled release, and high drug loading [118,119]. They have the main function of facilitating drug transport due to their ability to fluidize skin lipids [120]. Phospholipids, cholesterol, mono-, di- and triglycerides, fatty acids, waxes, and steroids are the most common lipids used in liposomes development. Surfactants such as, poloxamers and polysorbates can be used to enhance formulations stability [17]. Rosseto et al. [100] developed lipid nanoparticles to deliver propolis. In this study, nanoparticles loading propolis were administered in wounded skin and wound closure was quantified, confirming propolis potential in accelerating healing process after 15 days.

As demonstrated above, nanoparticles have several advantages, in addition to high carrier capacity, high stability, ability to incorporate both hydrophilic and hydrophobic materials, ability to use a variety of delivery methods [121], biocompatibility and skin tolerability [122], biodegradability, low toxicity [123], and low irritancy [124]. Although there are some disadvantages that may vary according to the type of nanoparticle. For example, the presence of permeation enhancers in nanoemulsions may compromise the integrity of the stratum corneum’s lipids [125], in addition to the difficulties of removing organic solvents from these compositions [124]. Another example is stability issues, difficulties with scale-up process [126], and high cost [127] for liposomes preparations.

#### 3.4.2. Polymeric Nanofibers

Different polymers can be used to produce fibers in the nanometric range and they are called nanofibers, presenting different final properties and potential applications. Examples of natural or synthetic polymers include collagen, cellulose, silk fibroin, poly(lactic acid) (PLA), polycaprolactone (PCL), polyurethane (PU), and poly(lactic-co-glycolic acid) (PLGA). Nanofiber dressings could be an alternative for chronic wounds by replacing natural provisional extracellular matrix until it is regenerated [17]. They also act in preventing bacterial contamination in the wounded area, forming a physical barrier, hindering microorganisms invasion [55]. Synthetic or natural active agents can be incorporated in nanofibers, enhancing its activity [128,129,130]. *Aloe vera* L. and recombinant human epidermal growth factor, for example were incorporated and results indicated that high concentrations of this active might be a suitable strategy for chronic wounds treatment [17].

Sangnim et al. [104] developed a clindamycin-loaded polymeric nanofiber patch composed of polyvinyl alcohol (PVA) and tamarind seed gum. Authors studied different concentrations of PVA, gum, and model drug to produce the polymeric nanofibers, adjusting the processing parameter in each case. Continuous fibers were obtained when using PVA concentrations between 10% and 15% (*w*/*v*), and fiber diameter as proportional to PVA concentration and inversely proportional to applied voltage (diameter decreased with lower concentrations and higher voltages). Clindamycin-loaded fibers inhibited *Staphylococcus aureus* growth more effectively than commercial clindamycin gel product. This nanofiber was later improved using Eudragit® S100 to form a bilayer patch, enhancing its durability and easiness of use [103].

Another example of polymeric nanofibers is bacterial nanocellulose, which has been studied for medical purposes, including its application in wound healing, due to its favorable properties [131]. Bromelain was also incorporated in bacterial nanocellulose membranes, leading to a system with higher antibacterial activity [12]. Nisin, an antimicrobial peptide synthetized by several microorganisms, was also incorporated in bacterial nanocellulose membranes, forming a stable system with antioxidant and antibacterial activity against Staphylococcus aureus, Escherichia coli, and Pseudomonas aeruginosa [101,102].

Among the advantages of polymeric nanofibers, their high protein adsorption rates, a crucial modulator of cell attachment to a biomaterial surface [132], similarity of membranes to the natural extracellular matrix permitting cell penetration, differentiation, and adhesion [133], good flexibility [134], high surface area to volume ratio favoring cell attachment [135], and drug loading stand out. One production method, self-assembly, has some limitations, such as high cost, low productivity, and complicated processing [136].

#### 3.4.3. Nanohybrids

Nanohybrids represent a combination of multiple nanostructures into one cohesive structure [23]. These could be achieved by combining nanoparticles and liposomes, or even nanoparticles in hydrogels or nanofibers.

Polyvinyl(alcohol)/chitosan/nano zinc oxide nanocomposite hydrogels were investigated regarding their potential use as dressing for wounds. In this case, hydrogel development parameters were studied to optimize conditions. Other important parameters, such as morphology, mechanical properties, toxicity, protein absorption, antibacterial activity, and in vitro wound healing, were analysed. The resultant hydrogel presented antibacterial properties, was biocompatible, showed no toxicity and in vitro potential to treat wounds [108].

Ding et al. [56] developed a new hydrogel material composed with chitosan crosslinked with genipin and *Bletilla striata* polysaccharide, which presented better properties than chitosan crosslinked only with genipin. However, this material did not show good antibacterial activity, and to overcome this issue, a nanohybrid was proposed by the incorporation of silver nanoparticles in the final formulation. The nanohybrid dressing provided gas permeation and water retention ability, supressed bacterial proliferation, and enhanced fibroblasts proliferation, showing great potential to be further to be used to promote wound healing.

Bacterial nanocellulose membranes have also been studied for the impregnation of nanoparticles to form nanohybrid systems [109,110]. Zinc oxide nanoparticles were successfully impregnated in bacterial cellulose membranes, and exhibited antimicrobial activity against *E. coli*, *P. aeruginosa*, *S. aureus,* and *C. freundii*. In a burn mice model, bacterial cellulose containing zinc oxide nanocomposites showed significant healing activity, with fine tissue regeneration proven by histological analyses when compared to bacterial cellulose [109]. In another study, bacterial nanocellulose membranes were immersed in a silver nitrate solution, with the posterior reduction of silver ion to the metallic silver nanoparticles. Authors showed that nanoparticle-impregnated membranes exhibited strong antimicrobial activity against *Escherichia coli* and *Staphylococcus aureus*, which can contaminate wound beads [110].

The benefits of nanohybrids include improved esthetic qualities, easy handling, low polymerization shrinkage, great polishability and durability [137,138,139], and the fact that they can combine different treatments to boost therapy effectiveness [23]. However, more studies on their toxicity are still needed [140].

## 4. Conclusions

Wound healing is a well-orchestrated process comprising four overlapping and dependent phases, which are regulated by cellular, humoral, and molecular mechanisms. This complex and intricate sequence occurs naturally but could be enhanced and accelerated by dressing techniques, products, and actives. Wound management has proven to avoid non-healing complications, and the use of topical chemotherapy has improved the survival of patients with major chronic wounds and burns. Over the past years, dressings have developed with the arrival of new alternatives, including mixtures of different polymers and nanotechnology tools to create improved materials while guaranteeing an optimal environment. Improvements in hydrogel manufacturing along with nanotechnology can provide new, versatile, and innovative technologies for the future of wound dressing and wound repair. Therefore, besides focusing on the enhancement of nanotechnology-dressings characteristics, researchers should consider the development of cost-effective products, aiming towards the improvement of patients’ quality of life and expenditure reduction.

## Figures and Tables

**Table 1:** Definition, main characteristics and advantages of pharmaceutical forms used in interactive and biological dressings.

Dressing Type	Definition	Main Characteristics	Advantages	Examples (References)
Alginate	Dressings made of calcium alginate, an anionic polysaccharide produced from brown seaweed	Calcium ions from dressing are exchanged with sodium ions from wound environment, forming a gel	Can absorb 15–20 times their weight in fluids; can be removed intact; considered long-term dressing	Release of therapeutic proteins [82]; containing chlorhexidine hexametaphosphate nanoparticles [83]; Sulfide-releasing property [84]
Films	Semipermeable dressings made from polyurethane and coated with an acrylic derivative adhesive	Transparent, gas and water vapour permeable	Allow easy wound monitoring (transparent dressing); can be changed only when necessary and removing cause simple and small trauma in wound region	Electroactive shape memory polyurethane-urea films [85]
Foams	Composed of polyurethane or silicone with a semi-occlusive outer layer	Outer layer is permeable to water vapour and serves as barrier for microorganisms’ infection, polyurethane center absorbs exudate	Able to create or maintain a moist environment; easy and nom-traumatic removal; can absorb and retain wound exudate	Hemostatic polyurethane-urea foams [86]
Hydrocolloids	Crosslinked polymer matrices with integrated adhesives and starches, such as cellulose, gelatin, pectin and guar	Occlusive and adhesive dressing, which form a gel upon contact with wound exudate, permeable to water vapour, allow debridement	Form gels in contact with wound exudate; capacity to promote wound debridement; long wear-time	*Centella asiatica* loaded hydrocolloid based on sodium alginate [60]
Hydrofiber	Contain carboxymethyl cellulose formed into textile fibers	Highly absorbent fibers, form gels upon exudate absorption, allow autolytic debridement	Can absorb 25 times its own weight; form gel when in contact with wound exudate; encourage autolytic debridement	Hydrofiber dressing with silver [78]
Hydrogels	Water-based products, designed as polymeric networks, comprised of up to 96% water	Clear to transparent, capable to absorb biological fluids, permeable to water and oxygen	Allow easy wound monitoring (transparent dressing); capable of absorbing biological fluids; maintain the area moisturized; promotes autolytic debridement; help cell proliferation and epithelization process; minimal or null trauma in their removal; permeable to water and oxygen	Hydrogels of PNIPAAm-co-Aam to release bromelain [87]; triple polymer hydrogel (chitosan, gelatin and PVA) loaded with moxifloxacin [88]

**Table 2:** Summary of preparation process used to develop reviewed nanotechnology-based systems used for wound healing.

Nanotechnology-Based System	Description	Material	Active Loaded	Preparation Process	Study Type (References)
Nanoparticles	Inorganic nanoparticles	Silver and gold	None	Phytochemical assisted thermal reduction	*In vivo* [92]
		Silver	None	Phytochemical assisted thermal reduction	*In vitro* [93] and *in vivo* [94]
		Zinc oxide	None	Room temperature synthesis and solvothermal synthesis	*In vitro* [95]
	Polymeric nanoparticles	Chitosan	Silver sulfadiazine	Ionotropic gelation	*In vitro* [96]
			Bromelain	Ionotropic gelation	*In vitro* [97,98,99]
	Solid lipid nanoparticles	Poloxamer 188 and tristearin	Propolis	Stirring followed by ultrasonication	*In vitro* and *in vivo* [100]
Polymeric nanofiber	Composite bilayer film	Polyvinyl alcohol (PVA) and gelatin/chitosan/polyethylene glycol (PEG) blend	None	solution casting and crosslinking agent	*In vitro* [66]
	Membrane	Bacterial nanocellulose	Bromelain	Bacterial cultivation	*In vitro* [12]
			Nisin	Bacterial cultivation	*In vitro* [101,102]
	Nanofiber	Polylactic-co-glycolic acid (PLGA)	Recombinant human epidermal growth factor and *Aloe vera* extract	Electrospinning	*In vitro* and *in vivo* [17]
		Polyvinyl alcohol (PVA) and tamarind seed gum	Clindamycin	Electrospinning	*In vitro* [103,104]
Nanohybrids	Hydrogel and nanoparticles	Alginate and gellatin hydrogel	Silver nanoparticles	Homogenization with mechanical stirrer	*In vitro* and *in vivo* [105]
		Chitosan hydrogel	Zinc oxide nanoparticles	Nanoparticles: reduction with NaOH Hydrogel: pH change of chitosan solution Nanohydrid: homogeneization of NP and hydrogels followed by freeze-drying	*In vitro* and *in vivo* [106]
		β-chitin hydrogel	Zinc oxide nanoparticles	Nanoparticles: reduction with NaOH Hydrogel: crosslink with CaCl_2_ Nanohydrid: homogeneization of NP and hydrogels followed by freeze-drying	*In vitro* and *in vivo* [107]
		Polyvinyl alcohol (PVA) and chitosan	Zinc oxide nanoparticles	Freeze-thaw method	*In vitro* [108]
		Chitosan and *Bletilla striata* polysaccharide	Chitosan-Ag nanoparticles	Hydrogel sponge: homogeneization followed by freeze-drying Nanoparticles: reduction followed by freeze-drying Nanohybrid: nanoparticles were crosslinked with genipin and frozen, followed by freeze-drying with sponges	*In vitro* and *in vivo* [56]
	Membranes and nanoparticles	Bacterial nanocellulose membranes	Zinc oxide nanoparticles	Membranes: bacterial cultivation Nanohybrid: impregnation of ZnO NP	*In vitro* and *in vivo* [109]
		Bacterial nanocellulose	Silver nanoparticles	Membranes: bacterial cultivation Nanoparticles: silver nitrate reduction with sodium borohydride Nanohybrid: impregnation of silver nitrate	*In vitro* [110]
	Nanofiber and nanoparticles	Polycaprolactone nanofibers	Zinc oxide nanoparticles	Electrospinning	*In vitro* [111]

## Data Availability

Not applicable.
